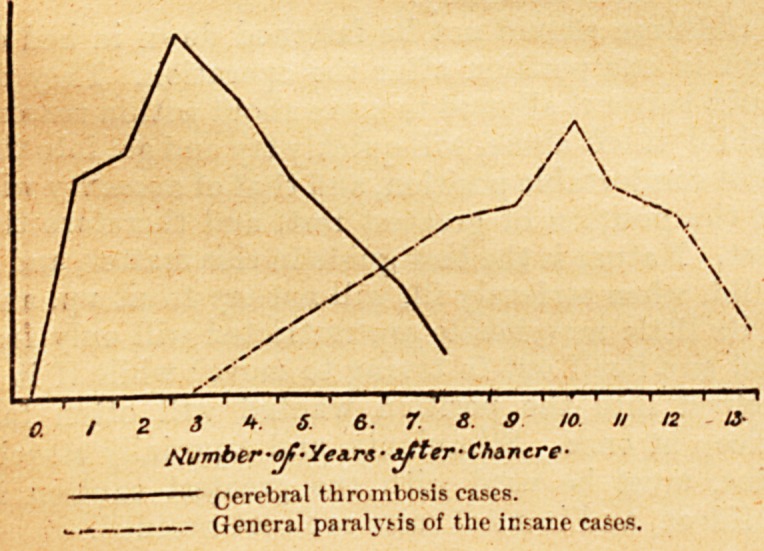# Paralysis and Syphilis

**Published:** 1907-03-02

**Authors:** 


### Points in Treatment.

### PARALYSIS AND SYPHILIS.

### The Time after the Primary Chancre at which Cerebral Syphilis and General Paralysis of the Insane are most Liable to Occur.

Cerebral syphilis may occur within a year of the primary sore; on the other hand, it may be postponed for forty years. The question arises: At what time is it most likely to occur? Is it more likely to occur late or early? Professor Fournier's analysis of 223 cases of *cerebral syphilis* with hemiplegic result shows most clearly that the liability to this form of cerebral syphilitic affection is *at its height three years after the chancre*, and that the great majority of the cases occur within seven years of infection. Out of the 223 cases—

25 occurred within 1 year of the chancre.
26 occurred between 1 and 2 years after the chancre.
39 occurred between 2 and 3 years after the chancre.
29 occurred between 3 and 4 years after the chancre.
20 occurred between 4 and 5 years after the chancre.
9 occurred between 5 and 6 years after the chancre.
13 occurred between 6 and 7 years after the chancre.

*i.e.*, 161, or 72 per cent., within seven years after the chancre.

A similar analysis of 112 cases of *general paralysis of the insane* shows that the tendency was *at its height during the tenth year after the chancre.* No case occurred during the first or second years, whilst there were increasing numbers each subsequent year up to the tenth, after which there was a rapid decline. Thus—

During the seventh year after the chancre there were 8 cases of general paralysis of the insane.
During the eighth year after the chancre there were 10 cases of general paralysis of the insane.
During the ninth year after the chancre there were 10 cases of general paralysis of the insane.
During the tenth year after the chancre there were 15 cases of general paralysis of the insane.
During the eleventh year after the chancre there were 12 cases of general paralysis of the insane.
During the twelfth year after the chancre there were 11 cases of general paralysis of the insane.
During the thirteenth year after the chancre there were 5 cases of general paralysis of the insane.

*i.e.* 64 per cent. of the cases occurred between seven and thirteen years after the chancre. The dates at which syphilitic thrombosis and general paralysis of the insane respectively are most likely to occur are therefore very different from one another; perhaps the accompanying diagrammatic curves may illustrate this more clearly still.

It is not those cases in which the primary and secondary symptoms are most severe that are most liable to be followed by cerebral syphilis or by general paralysis; these may follow even though the earlier symptoms were quite slight. It is possible that this may depend upon the less radical treatment that the slighter cases are liable to receive. If there is one thing certain about syphilis it is that if later troubles are to be prevented, treatment must be persevered with for a long while after the chancre, and this applies as much to the mild cases as to the severe. The common practice is to persevere assiduously with mercurial treatment for two years after the last noted symptom, and then to desist in the belief that the cure is complete. Cerebral syphilis and general paralysis, however, may occur in spite of this, and, owing to the maximum liability to parasyphilitic affections (tabes dorsalis, G. P. I.) beginning about the sixth year and being greatest about the ninth or tenth years, Professor Fournier lays stress on the necessity to repeat the mercurial treatment in the fifth and in the eighth years, especially in cities, in which, owing to business activity and anxiety, with consequent wear and tear of the brain, general paralysis of the insane is more likely to occur than it is elsewhere. The leading French authorities suggest that, subject to modifications according to the necessities of each particular case, the course should be as follows:—

During the first and second years, vigorous mercurial treatment with short intervals.
During the second and third years, therapeutic repose.
During the fifth year, vigorous mercurial treatment again.
During the sixth and seventh years, therapeutic repose.
During the eighth year, vigorous mercurial treatment again.

After this no further treatment, unless some special occasion should arise, is needed. It seems worth while to bring this French scheme of treatment before English practitioners, for it is far more radical than that which is usually adopted in England. It is to France that many English patients are sent for the cure of late syphilitic affections, and French methods may well be adopted here.

It may further be remarked that the French do not regard potassium iodide as a preventive of late symptoms of syphilis; potassium iodide will cure gummata very readily, but it is well known that gummata may develop even during the administration of potassium iodide. The iodide will put them away again, but few syphilitic affections other than actual gummata are cured by potassium iodide. The French have little or no faith in the drug as a preventive; it is upon mercury that they mainly rely. After mercury has once been given in quantity, there is evidence to show that much of it remains stored in the bones. The giving of iodide may liberate some of this mercury, in which case the patient is virtually being given mercury though there is only potassium iodide in his medicine. Nevertheless the French teaching is that whether potassium iodide be given or not, there is no stage of syphilis at which it is too late to administer mercury.

## Figures and Tables

**Figure f1:**